# Editorial: Emerging trends in biosensors: bridging chemistry and practical applications

**DOI:** 10.3389/fchem.2026.1847404

**Published:** 2026-04-23

**Authors:** Rui Wang

**Affiliations:** 1 Center for Medical Research and Innovation, Shanghai Pudong Hospital, Human Phenome Institute, State Key Laboratory of Genetics and Development of Complex Phenotypes, School of Life Sciences, Fudan University, Shanghai, China; 2 International Human Phenome Institutes, Shanghai, China

**Keywords:** biosensors, chemical design, practical applications, rational design, sensing mechanisms

The intersection of chemistry and biosensors has become one of the most dynamic and fast-growing fields in modern analytical science, with profound impacts on disease diagnosis, pathogen detection, environmental monitoring, and biomedical research. In recent years, innovative strategies including molecular probe design, electrochemical interface engineering, CRISPR-mediated signal amplification, and near-infrared optical sensing have greatly improved the sensitivity, specificity, stability, and portability of biosensing systems. However, challenges still remain in achieving highly reproducible surface preparation, reliable signal output, straightforward readout, and seamless translation from laboratory prototypes to real-world applications. To highlight the latest progress toward solving these key issues, we are pleased to present this Research Topic “Emerging Trends in Biosensors: Bridging Chemistry and Practical Applications”. This collection brings together cutting-edge studies covering electrochemical electrode pretreatment, near-infrared fluorescent probe mechanisms, CRISPR-powered naked-eye biosensing, and metal-complex-based bioactive sensing systems. Together, these works emphasize the critical role of chemical design and mechanism exploration in building high-performance biosensors, and provide new insights for developing robust, user-friendly, and practically applicable diagnostic tools for real-world.

The first article by Yuan et al. is dedicated to the development of a naked-eye biosensing system for *Mycobacterium tuberculosis* (MTB) by integrating one-pot RPA amplification, CRISPR/Cas12a cleavage and G4-hemin DNAzyme self-assembly. Glycerol is used as a phase-separation barrier to avoid mutual interference between RPA and CRISPR/Cas12a, enabling efficient one-pot reaction. Upon target recognition, Cas12a cleaves the ssDNA reporter and triggers the formation of active G4-hemin nanozymes, which catalyze TMB to produce a visible blue color signal. The system achieves ultrasensitive visual detection of MTB with a limit of detection of 10 copies/μL within 60 min without complex instruments. Clinical validation with 104 patient samples shows 100% concordance with standard methods, demonstrating high specificity, speed, and practicality for point-of-care MTB testing in resource-limited settings.

The second article by Zaki et al. focuses on the synthesis, solution equilibrium characterization, and anticancer/antimicrobial activity evaluation of platinum-group metal half-sandwich complexes with 1-(α-D-glucopyranosyl)-4-hetaryl-1,2,3-triazole ligands. The study explores how the α-anomeric configuration of the glucose unit affects the complexes’ biological performance by preparing a series of Ru(II), Os(II), Ir(III), and Rh(III) complexes via ligand coordination and characterizing their structures through X-ray crystallography and potentiometric titration. The results reveal that the α-configured complexes maintain potent and selective cytostatic activity against a broad range of cancer cell lines (including cisplatin-resistant ovarian cancer cells) at low micromolar to submicromolar IC50 levels, acting through oxidative stress induction, while their bacteriostatic activity against multidrug-resistant Gram-positive bacteria (MRSA, VRE) is weaker than that of the β-anomeric counterparts.

The third article by Khor et al. is dedicated to investigating the electropolishing pretreatment of Metrohm BT220 gold screen-printed electrodes (SPEs) using electrochemical techniques including cyclic voltammetry (CV) and electrochemical capacitance spectroscopy (ECS). This study clarifies how CV parameters such as cycle number, scan rate, and potential upper limit affect electrode surface area, reproducibility, and stability. They showed that controlling CV cycles to reach a consistent gold reduction peak current yields highly reproducible surfaces. Furthermore, the integrated Ag pseudo-reference electrode is unstable in ferricyanide/ferrocyanide redox solutions, and the electrode is damaged by ethanol, a common solvent for thiol modifiers. These findings highlight the critical importance of optimized pretreatment for reliable biosensor development.

Final article by Zhang et al. is dedicated to revealing the fluorescence detection mechanism of NIR fluorescent probes based on intramolecular spiro cyclization by means of quantum chemical calculations. This theoretical approach helps to analyze the electronic structure, π-electron distribution, and electron transfer characteristics of three representative probes (NIR-pH, NIR-ATP, NIR-Hg) targeting H^+^, ATP, and Hg^2+^ respectively. They showed that the probes stay in a weak fluorescence state under the closed spiro structure due to interrupted π-conjugation, while the ring-opening reaction triggered by target analytes recovers continuous π-electron delocalization and switches the excitation mode to local excitation, thus realizing significant fluorescence enhancement. Furthermore, the calculated spectral parameters are highly consistent with experimental values, which validates the rationality of the cyclization/ring-opening sensing mechanism and provides a theoretical basis for the rational design of high-performance NIR fluorescent probes.

These four articles collectively point to a core insight: chemistry is not merely a tool for biosensors, but rather their intrinsic determinant. The performance of each sensor—sensitivity, specificity, response speed, and stability—is ultimately governed by the controllability, reversibility, and efficiency of the underlying chemical reactions. More importantly, these studies all follow a similar structure-activity relationship paradigm: by systematically varying chemical parameters and precisely measuring their impact on sensor performance, researchers are advancing the development of biosensors from a “trial-and-error” approach toward “rational design”. Thus, within the interdisciplinary field of biosensors, chemistry serves as the converter between the biological world and physical signals, and as the ultimate foundation for achieving high-precision and high-reliability detection.

We sincerely thank all distinguished reviewers for their timely and rigorous reviews of the manuscripts, despite their busy schedules. Their professional contributions have greatly improved the quality of this Research Topic. We hope the contents of this Research Topic will prove valuable and contribute to the development of related research in this field.

